# Factors influencing physical activity and sedentary behaviour in contact centres during the COVID-19 pandemic and their relevance for the future of hybrid working

**DOI:** 10.1371/journal.pone.0312473

**Published:** 2024-10-23

**Authors:** Jillian Manner, Divya Sivaramakrishnan, Graham Baker, Scott Lloyd, Ruth Jepson

**Affiliations:** 1 Scottish Collaboration for Public Health Research and Policy, University of Edinburgh, Edinburgh, United Kingdom; 2 Physical Activity for Health Research Centre, University of Edinburgh, Edinburgh, United Kingdom; 3 Public Health South Tees, Middlesbrough Council and Redcar & Cleveland Borough Council, Middlesbrough, United Kingdom; 4 Fuse–UKCRC Centre for Translational Research in Public Health, Population Health Sciences Institute, William Leech Building, Newcastle University, Newcastle upon Tyne, United Kingdom; 5 Teesside University, Middlesbrough, United Kingdom; University of Central Lancashire, UNITED KINGDOM OF GREAT BRITAIN AND NORTHERN IRELAND

## Abstract

**Background:**

The COVID-19 pandemic changed the way many industries work, including contact centres, with some employees working from home and new on-site restrictions/measures in place representing even greater challenges for employers around staff engagement and wellbeing. This study aimed to understand the interplay of individual, social, environmental and organisational factors influencing physical activity and sedentary behaviour in UK contact centre employees, how the pandemic impacted these factors, and their relevance for the future of hybrid working.

**Methods:**

Individual interviews (n = 33) were conducted with participants (staff working full and part time, on site and from home) from four UK contact centres. A topic guide based on the ecological model was developed to understand current barriers and facilitators to physical activity and (reducing) sedentary behaviour during and outside of working hours. Thematic analysis was carried out using a codebook and a deductive coding approach to identify themes.

**Results:**

Three key insights are provided. First, participants felt they were generally sitting more and moving less since the first UK-wide lockdown. Second, factors which negatively impacted on these behaviours were evident across all levels of the ecological model. These included individual and social barriers (e.g., lack of motivation and preferable physical activity options) as well as environmental and organisational barriers (e.g., poor home office setup, back-to-back virtual meetings). There were a mix of new and existing barriers (exacerbated by the pandemic) and several of these were linked to homeworking. Third, organisational support requirements (e.g., homeworking ergonomic support) and existing facilitators (such as the provision of informational support and flexible working arrangements) were identified.

**Conclusion:**

Solutions to reduce sedentary behaviours and increase physical activity in contact centres need to address barriers from the individual to the organisational level. Whilst the study was undertaken in the UK, the results are like to be applicable globally.

**Trial registration:**

**Clinical trial registration:** The trial for the wider project has been registered on the ISRCTN database: http://www.isrctn.com/ISRCTN11580369.

## Introduction

The definition of sedentary behaviour is widely adopted as “any waking behaviour characterized by an energy expenditure of ≤1.5 METs, while in a sitting, lying or reclining posture” [1]. Multiple meta-analyses and systematic reviews provide evidence that high levels of sedentary behaviour increases the risk of a host of negative short and long term health outcomes, such as the development of certain cancers, cardiovascular disease, diabetes, various musculoskeletal conditions, depression and premature mortality [2–6]. Conversely, physical activity behaviours involve an energy expenditure of >1.5 METs [7]. Although often considered two distinct behaviours, recent studies demonstrate a complex relationship where physical activity can modify the associations between sedentary behaviour and the associated risks of various chronic conditions and mortality [8–10]. The current global physical activity guidelines recommend that adults aim for 150 to 300 minutes of moderate or 75 to 150 minutes of vigorous aerobic physical activity per week, and to limit the time spent in total and prolonged periods of sedentary behaviour [11]. Given these definitions, one may choose to position the two behaviours on the same spectrum of “movement” according to their respective energy expenditures, but in separate (opposing) locations [1]. Thus, interventions need to specifically tailor to a specific part on the same spectrum. The distinction between these behaviours in terms of definitions, guidelines and also interventions is still important, as evidence shows that interventions aimed at reducing sedentary behaviour rather than increasing physical activity were most effective at reducing sitting time [12]. As such, it is important to address sedentary behaviour directly, rather than assume it is being tackled indirectly through other means such as during attempts to increase physical activity.

Occupational sitting contributes significantly to overall daily sitting. For example, in the United Kingdom (UK), office workers spend between four and nine hours (81%) of their work day sitting and an average of 67 days per year [13]. According to a 2014 cross-sectional study, workday sitting time in the UK accounted for 54% of total daily sitting time [14]. Given that sedentary behaviour and physical activity are so closely intertwined, it is unsurprising that occupational physical activity (including commuting) has decreased over time [15–17] and that this has contributed to an overall decline in physical activity globally [16, 17].

A defining feature of ecological models is the specification that intrapersonal variables, interpersonal and cultural factors, and physical environments can all influence behaviour [18]. This perspective suggests that sedentary and physical activity behaviours during the workday are influenced by multiple factors operating at different levels [18] including organisational and societal factors such as workplace social norms, workload [19, 20] and built environment factors such as the layout of the office [21]. Considering the variety of risks associated with frequent and prolonged occupational sedentary time, ways of working which facilitate such unhealthy behaviours place a large burden on employees, employers and healthcare systems and the wider economy.

Sedentary behaviour is particularly high, and physical activity levels low in contact (call) centres in comparison to other office-based workplaces [22, 23]. For example, objectively measured data from one contact centre in Australia indicated that staff spent 83.4% of their work time sedentary, in comparison to 75.8% and 73.7% for general office and customer service staff respectively [22]. A similar study in Sweden found that contact center staff spent 80% of their shift seated [24]. Contact centres are often tightly controlled worksites where employees are strictly supervised and required to maintain high levels of productivity [14], and as such employees may have less autonomy to move around and stand [23]. Due to these and other organisational and environmental factors, contact centre staff often have poorer physical and mental health outcomes [22, 23, 25]. These findings were found to be independent of education in one study [25], although call handlers are less likely to have completed further education according to another [22].

Factors which influence health behaviours are often understood by categorising them by contextual factors or ‘levels’ (individual, social, environmental, organisational) derived from the ecological model of behaviour [26]. For example, the model has previously been applied to work-related sedentary behaviour and physical activity, indicating that these behaviours are influenced by a variety of factors [18] including individual (e.g. personal), social (e.g. workplace social norms), organisational (e.g. workplace social norms and workload) [19, 20] and environmental (e.g. the built environment in and around the workplace) [21].

As with other traditionally office-based organisations, the contact centre industry had to grapple with transitioning to a hybrid working model, which was necessitated by the onset of the COVID-19 pandemic in 2020. There is emerging evidence this transition has had a deleterious effect on both physical and mental health outcomes for office workers [27–32]. Demands of the home working environment, levels of organisation support and social connections outside of work are all factors which may contribute to the impact of hybrid and home working [27, 30]. Thus, office workers were subjected to additional barriers, unique to the pandemic context, to decreasing their sitting time and increasing physical activity behaviours. Understanding the unique contextual factors related to sedentary and physical activity behaviours in contact centre staff during the pandemic is needed in order to effectively intervene and create sustainable change. Given the complex relationship between physical activity and sedentary behaviour and the associated influential factors and risk factors, it is important to investigate their specific barriers and facilitators in the same instance, whilst keeping in mind that they are distinct behaviours. Investigating these barriers and facilitators within the context of the ecological levels allows for a more in-depth understanding of the most dominant factors that impact on sedentary and physical activity behaviours. Few studies investigate sedentary behaviour and physical activity together, using the ecological model and with both home and in-office workers. Drawing on this model, the current article investigates how changes to work during COVID-19 and the consequent lockdown influenced physical activity and sedentary behaviour among contact center workers. The study offers a novel investigation of the obstacles and opportunities connected with this new work climate in the context of physical activity and sedentary behaviours using a model which pinpoints the dominant behavioural influences. By investigating these health behaviours in the new hybrid work context, and applying them to an already struggling industry, this study extends prior literature on workplace health behaviours and workplace social norms.

The overall aim of this study was to investigate how the COVID-19 pandemic influenced the modifiable determinants of workplace physical activity and sedentary behaviour in contact centre employees and their relevance for the future of hybrid working. The research questions were as follows:

1. How did/does the COVID-19 pandemic impact on health behaviours during different working conditions?
2. What are the specific barriers and facilitators to physical activity and (reducing) sedentary behaviour among staff during the COVID-19 pandemic?
3. How can these barriers and facilitators be contextualised using the ecological model to understand behavioural influences at various levels?

## Methods

### Study design

The current study is qualitative and involved semi-structured interviews. The qualitative design was chosen to understand experiences and perceptions of staff regarding the barriers and facilitators to sedentary behaviour and physical activity during the COVID-19 pandemic and interviews were semi-structured to give participants the freedom to express views on topics which may not have been pre-determined by the researchers [33]. Additionally, the novel combination of factors being investigated (physical activity and sedentary behaviour during the pandemic and in the context of the ecological model) made it advantageous to gather in-depth insights rather than more rigid quantitative data. Acknowledging recent debates on the appropriateness of quality checklists [33, 34], the Consolidated criteria for Reporting Qualitative research (COREQ) checklist was used to guide the reporting of the data [35]. See S1 File for the completed checklist.

### Setting and sampling

The Stand Up for Health (SUH) intervention was developed by researchers from the University of Edinburgh with the aim of reducing sedentary behaviour in contact centres [36]. It used the 6SQuID intervention development framework, an adaptive programme for developing complex health interventions that considers and targets contextual factors [37]. From April 2019 to March 2021, a multi-centre, feasibility (stepped-wedge cluster RCT) study and process evaluation of SUH was conducted with funding from the National Institute for Health and Care Research (NIHR; PHR project grant 12/149/19). The development of the initial project ideas and grant proposal was undertaken whilst receiving funding for the Scottish Collaboration for Public Health Research and Policy through MRC Grant MR/K023209/1 (https://www.ukri.org/councils/mrc/). This evaluation will be referred to as the SUH project throughout the paper. The protocol and results from the SUH project has been published separately [38, 39]. During the COVID-19 pandemic (between July and September 2020) and first UK lockdown period, the SUH project team conducted online interviews with individual staff to understand their perceptions, needs and preferences regarding physical activity and sedentary behaviour during the pandemic. Four of the six contact centres from across England and Scotland who were participating in the post-lockdown phase of the SUH project opted to take part in the current study (two each from the public and private sectors). All staff from these four contact centres were given the opportunity to take part.

### Recruitment

Recruitment of participants took place between June 1st and August 31st 2020. To recruit contact centre staff, the researchers co-ordinated with contact centre stakeholders whom they were already in contact with through the SUH project. They provided stakeholders with a recruitment poster and participant information sheet which were sent to staff internally via email. Stakeholders also promoted the programme through word of mouth. The interviews were made available to everyone in each contact centre, however some staff were unable to access the Microsoft Teams platform used to conduct the interviews. In three of the centres (Centre A, B and D), the research team co-ordinated with the centre stakeholders who scheduled the interviews with interested staff members. In one centre (Centre C), a Doodle poll was used to schedule interviews directly with participants. Participants were not incentivised to participate.

### Data collection procedures

Interviews were conducted by two of the researchers (DS, JM) who are experienced in qualitative interviewing and in working with contact centres. For each interview, one researcher led while the other moderated, and they alternated roles after each one. Both researchers took field notes. Interviews took place online using Microsoft Teams between July and September 2020, were audio and video (optional) recorded and took approximately 20 minutes each. Following interview completion, audio recordings taken via Microsoft Teams were transcribed anonymously by a third party.

In line with the study objectives, a semi-structured topic guide was developed by the research team (S3 File). The topic guide aimed to capture the unique working arrangements of participants, as well as their physical activity and sedentary behaviour levels and the factors influencing them during the COVID-19 pandemic. The guide included specific questions pertaining to barriers and facilitators to sedentary behaviour and physical activity at the various ecological levels (environmental, social, individual). See Table 1 below for example questions.

**Table 1:** Example interview questions.

Section	Example Questions
Part 1: Current work conditions	1. Let’s start with your current work conditions. Where are you currently working from? 2. Tell us more about your work environment.
Part 2: Sedentary behaviour and physical activity	1. Are your levels of sedentary behaviour more, less or the same as before the pandemic? Why? 2. What are some of the current barriers you face relating to sitting behaviour?
Part 3: Support	1. What support, resources or tools would be helpful to overcome the barriers you mentioned?
Part 4: Activity preferences	2. On a scale of 1–10, how interested are you in the following activities, 10 being very interested and 1 being not at all interested. *[activities then listed for participant based on ecological level]*

### Data analysis

Interviews were analysed deductively using a codebook thematic analysis approach (as defined by Braun and Clarke, 2021) [40]. This more structured thematic analysis method was chosen, rather than for example reflexive thematic analysis, given the a priori decision to categorise factors based on the underpinning ecological model. Pre-determined codes aligned with the research objectives, including codes based on the ecological levels, were created and organised in a coding framework created by the research team. Transcripts were coded deductively based on this framework to develop themes. Coding was shared across three researchers (DS, JM, GB). Five transcripts were triple coded, two transcripts were double coded, and the remaining 26 transcripts were single coded in NVivo 12 data analysis software (QSR International). Multiple coders were used to seek “interpretative depth” [33], rather than as a means of increasing coding reliability or establishing consensus between coders. In fact, it is argued that inter-rater reliability is unable to appropriate units of analysis and produce theory-free knowledge, and as such rigor is not necessarily gained using this method [34].

### Ethics and informed consent

Ethics approval was received from the School of Health in Social Science Ethics Committee at the University of Edinburgh (Ref:STAFF142). Participants were asked to provide written informed consent using an online survey form hosted by Qualtrics [41] prior to the interview, and were also asked for verbal consent (in front of the researchers) before they began the interview recording.

## Results

### Participant characteristics

Interviews were conducted with 33 participants across four contact centres (two private, e.g., telecommunications and two public, e.g., government). The centres operating and working hours varied. For the two public centres: one was a 24-hour service, and the other operated Monday to Friday from 9am - 5pm. For the two private centres: one advertised shifts Monday to Friday from 8am - 6pm, and the other indicated that their contact hours were Monday to Sunday 7am—10.30pm (messaging online only), Monday to Friday 8am - 9pm, and weekends and bank holidays 8am - 8pm.

Seventeen participants were female and 13 were male. Seventeen participants worked full time, five participants worked part time, and eleven did not disclose if they were part time or full time. Eleven participants were working in the office (onsite), and 22 were working from home (WFH) (see Table 2 below). As data were collected during one of the UK lockdowns, the participants who went into the office did so because they could not do their job from home (as is/was common for contact centres where staff needed to have secure calls for example). As such participants either worked from home or onsite, but not both (hybrid). Participants are described in the text by their participant number, work location (on site or WFH) and hours (full time or part time). See S2 File for detailed participant characteristics.

**Table 2:** Work setting.

Work setting	Female	Male	Total
**Work from home**	11	11	22
**On site**	6	5	11
**Total**	17	16	33

The main results are presented here under three hierarchical themes: 1) changes to, and perceived consequences of, physical activity and sedentary behaviour levels, 2) influential factors (individual, social, environmental and organisational) and 3) organisational support required. Influential factors are broken down into four sub-themes; individual factors (namely anxiety, lack of motivation and work engagement), social factors (namely social restrictions at work and home, and those relating to online communication), environmental factors (namely reduced opportunities for incidental movement, closer of physical activity facilities and poor home office setup) and organisational factors (namely busyness with work, short breaks, and back-to-back virtual meetings).

### Changes physical activity and sedentary behaviour levels and consequences

The majority (n = 22) of participants described an increase in work and non-work- related sitting behaviour as compared to pre-pandemic. Of this group, those who worked from home found themselves taking fewer breaks and they all felt they were generally moving less during the workday. Four participants said their sitting time during the workday had decreased compared to pre-pandemic, mainly due to having more flexibility with breaks (and being able to do housework during them) if working from home, having more space to move compared to the office due to social distancing. Seven participants said their sitting time during the workday was similar to pre-pandemic. They felt this was because their jobs were the same, and when working from home they made additional efforts to stand up (sometimes when on calls if possible) and take active breaks to make up for the lack of incidental movement (e.g. taking the stairs or walking to someone’s desk). A total of 11 participants described some sort of increase in physical activity, although mostly outside of work. These individuals too felt this was due to increased flexibility (if home) and participating in physical activities with their household. Nineteen participants mentioned a decrease in physical activity both during and outside of work time for reasons such as a lack of preferred physical activity options and a lack of motivation. Five participants said their physical activity was generally the same as before the pandemic, either because they were not particularly active before or because they were able to maintain their pre-pandemic physical activity routine in some way.

### Factors affecting physical activity and (reducing) sedentary behaviour

**Fig 1:**
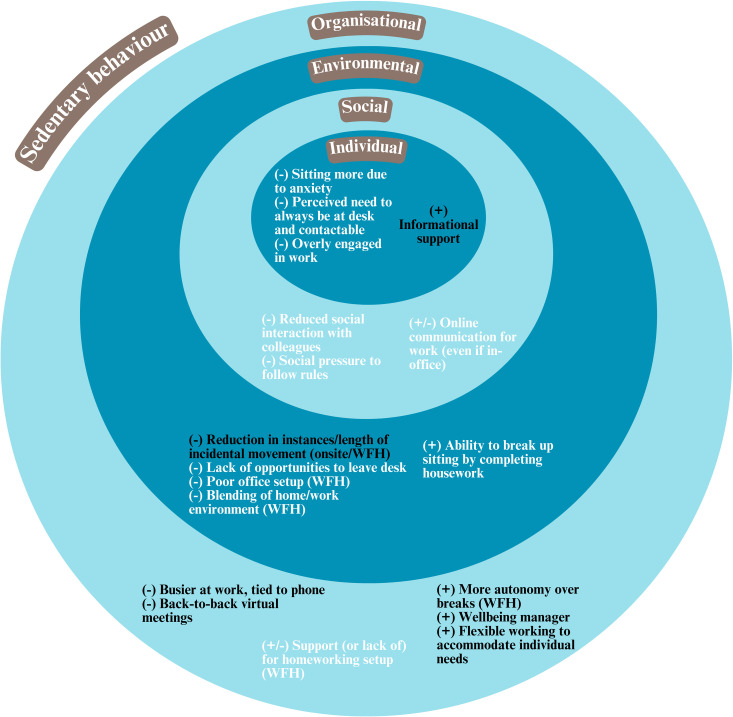
Summary of barriers (-) and facilitators (+) to reducing sedentary behaviour during the pandemic. (A) WFH means relevant to participants working from home, (B) Factors in black are related to both sedentary behaviour and physical activity.

**Fig 2:**
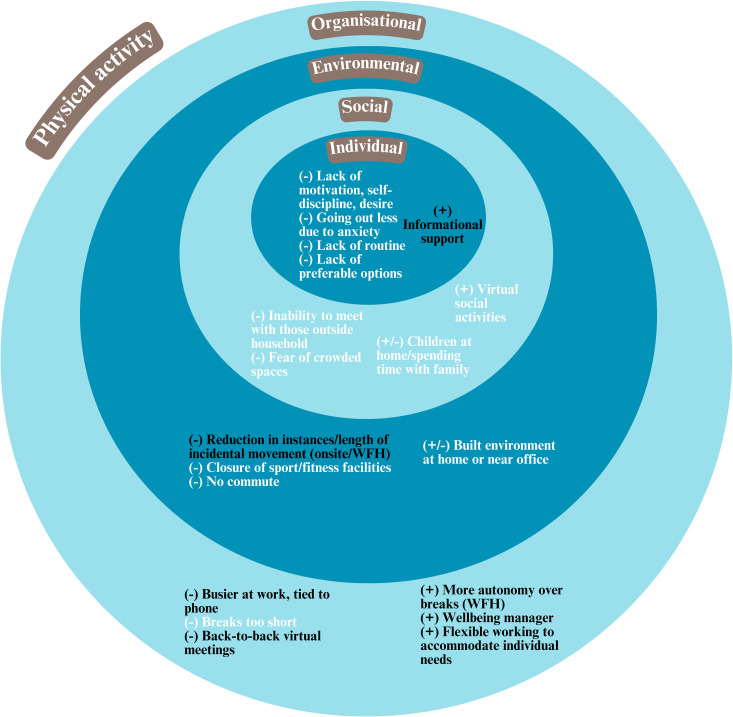
Summary of barriers (-) and facilitators (+) to physical activity during the pandemic. (A) WFH means relevant to participants working from home, (B) Factors in black are related to both sedentary behaviour and physical activity.

Participants described a range of interrelated individual, social, environmental and organisational factors (barriers and facilitators) to (increasing) physical activity and (reducing) sedentary behaviours while working from home and in the office during the pandemic. These are summarised in Figs 1 and 2, and described in detail below alongside quotes and examples which exemplify these factors.

#### Individual factors

Lack of motivation was the most prominent barrier to physical activity participation. Participants expressed a lack of self-discipline and desire to engage in physical activity before, during and after work. Participant 2 (male, onsite, full-time) explains:

“Getting out and getting the self-discipline and motivation to do it in lockdown has been really difficult for me…it’s basically go home [from the office after work] whilst this has been ongoing, and for the last three or four months I’ve virtually done no exercise at all.”

Many participants also cited a lack of motivation to break up sitting while working from home, namely because there were limited reasons and cues (such as walking to someone’s desk, going to the shops for lunch and commuting) to do so. This combination of individual barriers tied to motivation and physical opportunity meant that participants had to force themselves to take breaks, which proved challenging, especially when deeply engaged with a work task. This is contextualised in the quote below.

“In the office I suppose we would move around a lot because you would get up to get a drink or go to the vending machine or go to the kitchen. Or if your colleague…if you had to ask your colleague about a query, I would prefer to just stand up and go to their desk and speak to them. Whereas now obviously it always has to be through Skype or Teams, so I don’t have any need to move.” (Participant 11, female, WFH, full time)

A few participants mentioned they were struggling with pandemic-induced mental health challenges such as increased anxiety and low mood, which also impacted their motivation and desire to break up sitting and engage in physical activity. Participant 32 (female, onsite) describes her experience below.

“Before all of this [COVID-19], I was going out, doing more things, but with this going on, I have found myself, well, I’m working in a job where I’m sitting down…it has thrown my mood. Yeah, it’s not very good at the moment, but that’s something I will need to work on and push myself forward.”

Most participants described wanting to ‘stay in their bubble’, both in a social and environmental sense, to protect themselves from catching COVID-19. For participants who were going into work, fear of catching and spreading COVID-19 often meant that they stayed at their desks as much as possible, and continued to use online methods of communication, even if their colleagues were also in the office. Participants noted similar feelings towards home-working and opted to stay indoors and avoid unnecessary outdoor or social activities even if within the UK guidelines at the time.

Informational support provided by organisations, such as newsletters, and informational emails acted as a facilitator, as it often included information around sitting less and moving more.

Barriers specific to sedentary behaviour revolved around work engagement; participant’s perceptions of needing to always be at their desk when working from home (in case they were needed) and becoming immersed in work. One participant used her mobile phone for work, which meant she was always contactable and thus unable to truly take a break from work. These factors limited the frequency and duration of the breaks that participants felt they were able to take. Furthermore, working from home reduced participant’s psychological capability to stop working at the end of the day due the lack of prompts to do so (for example the office closing, others going home, having to catch a bus). This often led to longer working hours and more sitting, which Participant 9 (female, WFH) explains below.

“I forget to stand up to be honest, you’re just in that concentration of doing your job. Sometimes I think, oh my God, I haven’t stood up for three hours…whereas, in the office, you’re probably more likely to [stand up] because you get up to ask a question, you might get up to do something, or go to the toilet.”

Barriers specific to physical activity were related to a lack of preferred physical activity options and lack of routine during the pandemic. Most sporting facilities, gyms and other shared physical activity spaces were closed during the UK lockdowns, and outdoor group physical activity was not allowed or discouraged. Many participants noted that they were uninterested or unmotivated to take part in the limited activities that they were still allowed to do, such as in-home exercise, walking, running and cycling. Lack of routine, especially for those working from home who no longer had a commute or a reason to leave the house, was also cited. Below provides an example of these barriers.

“…before the pandemic, I was getting up every morning, five o’clock, going to the gym and things like that. With that not being an option to us anymore, I’m kind of using it as an excuse. So I just think, oh well, there’s no gym open anyway, I may as well have an extra couple of hours in bed. So I need to get myself in a routine basically.” (Participant 3, male, onsite, full time)

#### Social factors

Some participants described their work groups as being quite social, and that pre-pandemic they engaged in many group activities around the office which broke up sitting and provided opportunities to be active. Participants described social activities which increased physical activity during a normal workday onsite which were no longer viable due to the COVID-19 guidelines at the time of the interviews.

“…we were quite social as a work group and we would go for a walk every day at lunchtime. For around 40 minutes to an hour, we would walk into the local town, get lunch. Even if the weather was bad, we’d just do a few laps of the office.” (Participant 11, female, WFH, full time)

For participants who worked from home, all meetings and other colleague to colleague communication took place online (mainly through video calls, instant messages, and email) which meant they no longer walked to and from meetings rooms, offices, buildings, and others’ desks for formal and informal discussions. For participants who were still in the office, due to social distancing measures, they still tended to partake in a lot of online communication despite having the option to speak in person. For example, “you can’t go over to other peoples’ desks and have a bit of crack and banter with them, so it’s a little bit more difficult [to break up sitting]” Participant 5 (male, onsite). This led to an increase in sitting time and a decrease in incidental movement during the workday.

In the office, participants also felt social pressure to follow the rules and stay at their desk, utilising instant messaging and virtual calling if they needed help, “…rather than getting up and going to speak to the managers” (P4, male, onsite, part time).

*Social factors related to physical activity*. Many participants had a preference for participating in physical activity with others, both recreationally outside of work and during work time. Some had a preference for inherently social activities (team sports) and were not interested in activities such as walking and running, reducing their desire to engage in physical activity. Participant 13 (male, WFH, full time) explains:

“When I’m doing exercise, I want to be enjoying it. I want to be having my mind taken away from it, I don’t want it to be repetitive or anything like that. So, for example, if you go run on a running machine for an hour, you’re just there for an hour doing running and it’s just boring. If I’m running playing on the football pitch, you’re all over the pitch, you’re trying to do different things. That’s where I find enjoyment in the Monday night football. So I haven’t found anything or am aware of anything, something similar, to take my mind off, away, keep it busy, kind of thing.”

Others were open to activities that weren’t restricted, however lacked the motivation to do them alone. For example, Participant 19 (female, WFH, part time) said the following:

“…I have to make myself go for a walk or run. And it’s not the same when you’ve not got people…it motivates me when I’ve got people to run or walk with.”

A few participants took advantage of online exercise classes and other virtual fitness opportunities which helped facilitate physical activity behaviour during the pandemic. Some also mentioned that their organisations ran virtual social activities, like step count challenges. Participants also said they were socialising more with their family or those in their household, and sometimes engaging in physical activities with them. Participant 20 (WFH, full time) outlines this below.

“During the pandemic, physical activity for me has actually been quite good. My children are very sporty…so what we’ve done is probably Monday, Wednesday and Fridays we try to go for a family cycle after work.”

For some participants, having children at home was also seen as a barrier to physical activity participation, particularly if the majority of their free time during and outside working hours was spent on childcare. This led to an interplay of time, energy, stress and motivation related barriers. When asked about barriers to physical activity, Participant 22 (female, WFH) explained:

“…at the start [of lockdown], I was getting up early…to try and squeeze a little half hour thing [Les Mills virtual fitness class] in before I got ready for the day. But I think the whole lockdown experience of working pretty much full-time, having the kids at home, having a lot of work on, you just became physically exhausted and getting up early, even though it wouldn’t have been any earlier than you would have done prior to lockdown, just became a challenge. You would rather have the extra half hour in your bed because you just feel tired and at night, by the time we’ve got our children to bed, you just feel like you need to sit, you just don’t have the motivation to go, right I’m going to crack on and put a Les Mills class on and get on with it.”

#### Environmental factors

COVID-19 restrictions and the transition to primarily virtual communication reduced opportunities to break up sitting and for incidental movement. Some participants held roles which required them (when in the office) to frequently move around (for example if they were a manager who would check on staff at their desks). For these participants, the transition to homeworking led to a more significant increase in sitting and decrease in movement. Additionally, homeworkers often had much shorter distances to cover in their living areas as opposed to in the office, which meant that incidental movement (for example going to the toilet, getting a cup of tea, and going to the fridge) was shorter in distance and duration. Many participants also cited a lack of space when working from home as a barrier to moving more during the workday. Participant 22 (male, WFH, full time) explains:

“The walk to the toilet is shorter. The walk to get a cup of tea is shorter…you’ll maybe have more cups of tea if you’re in the office because you’ll have that longer conversation [with colleagues]. You’ll have a wander. You’ll have a chat with someone. Whereas [at home it is] just, put the kettle on, come back, that’s you done. There’s no other reason to be on your feet. That’s definitely a negative.”

Some participants who worked from home found the blending of the home and work environment led to longer working hours due to a difficulty in switching off, which increased sitting time. Some participants had unfavourable homeworking setups, such as unsuitable chairs, one screen (as opposed to the two or three they had in the office), no desk and other ergonomic challenges which made prolonged periods of sitting even less comfortable. Many participants were unable to stand as often due to wired headsets which limited their movement while on calls (also a barrier when in the office). Participant 9 (female, WFH, part time) described challenges with her homeworking setup below:

“The comfort of the chair [at home] is very much different. Even just sitting, with the computer screen, fitting everything on the desk. Usually, when I’m in the office, I use two computer screens, but at home I’ve only got the laptop. So, the position of the screen…it’s low down, rather than eye level… so positioning is different, it’s not comfortable if you’re here all day.”

A few participants felt that breaks were sometimes more flexible when working from home, and they often took them as opportunities to do housework, which got them to stand up and move a bit more than they otherwise would have.

For participants who worked from the office, they felt that new one-way systems governing movement around the workplace made it slightly more difficult to walk around, and they were often anxious about doing so due to COVID-19 and social distancing rules. Many social spaces were closed off or limited in such a way that severely limited social interaction within them. For example, Participant 3 (male, onsite, full time) said: “In the call centre, we’ve got a pool room. We’ve actually got table tennis as well, but at the moment, we’re not allowed to do anything like that.” In one centre during COVID-19, onsite employees had lunch “brought to your desk by catering staff rather than having to seek it” (Participant 4, male, onsite, part time). Although appreciated, this further decreased the need for leaving one’s desk area.

Homeworking participants stated they had fewer reasons to move around and go out especially when compared to having been in an office located near a town centre. No longer needing to commute often reduced physical activity, even if it had just required walk to the car or bus stop. Participant 10 (female, WFH) attributed this to “a lack of routine” saying, “I don’t need to walk to work, so again if it’s raining I haven’t got to leave the house. It’s harder to motivate myself to do that….I might get to Sunday and think, god I haven’t left the house in three days”.

However, being home did have some advantages. Some participants lived in areas with greenspace or blue space which encouraged them to be active outside. For example Participant 7 (male, WFH, full time) noted, “There’s better places to walk where I live, so I’ll do a 30-minute walk [on a break]. I wouldn’t normally do that where we work because it’s in the city centre and it’s not a very nice place to walk.” (P7, male, WFH, full time)

#### Organisational factors

Participants said that the nature of contact centre work lends itself to prolonged periods of sitting at a desk, especially in roles where the employee must be next to the computer for a prolonged period on calls. Breaks were sometimes hard to come by if there was a period of back-to-back calls that needed to be attended to. Some centres experienced an increase in call volumes during the pandemic, which made breaks even more difficult. Participant 9 (female, WFH, part time) emphasised this in saying, “the nature of the job is…you’re on the computer screen and it is mostly phone work. So, I’ve got to have access to the screen, and to be next to the computer for the phone calls.” However, one participant noted that they were encouraged to take their breaks whenever they could. The scheduling of back-to-back virtual meetings also presented barriers to break taking, and a few participants described experiencing ‘Zoom Fatigue’. Some participants had wireless headsets or earphones which allowed them to stand and move while on calls or in meetings.

Efforts to support home working setups for staff varied between centres. Although most participants felt they had the opportunity to have a Display Screen Equipment (DSE) assessment (a workstation assessment to identify risks associated with display screen equipment), not all took advantage of this, despite having an unsuitable working environment. Only some employees from some organisations were offered technical equipment (such as external monitors, screens, and mice) and even fewer were offered desk equipment (such as ergonomic chairs and desks). Unlike other companies, such as Google, who chose to give employees a $1000USD work-from-home allowance during the pandemic [42], almost none of the participants were offered such funds. Some participants felt that if they inquired about home working equipment, they would probably be able to get it, but chose not to due to lack of space or simply not wanting to ask.

The need to be desk bound for most of the day was often coupled with short break times. Participants noted that lunch breaks are usually only 30 minutes, which is often too short to engage in physical activity while still having time to eat and do other necessary non-work tasks such as use the toilet. Participant 3 (male, onsite, full time) describes this below:

“We get half an hour [lunch break]. So basically, we have time to…walk round the centre maybe, but…you don’t really have much time to eat your food and exercise…”

However, as noted above, some participants felt they had more autonomy over their breaks and thus an increased sense of flexibility when working from home and as a result of post-pandemic policies to support work-life balance. This mainly applied to managers, who, unlike call handlers, had more control over their schedule. Although, some call handlers did feel that they were not being monitored as much while working from home, which increased positive feelings of autonomy. One participant said her role had changed due to the pandemic, which meant that instead of sitting at her desk all day, she needed to be up and about to assist call handlers which meant she was on her feet most of the day.

A few participants mentioned they had a designated person (such as a wellbeing manager) who co-ordinated wellbeing activities and support for staff and acted as a facilitator for physical activity and (reducing) sedentary behaviour. Participants were also encouraged to reach out to their line managers if they needed support. One centre also had a helpline for staff who were struggling. Participant 1 (female, onsite, part time) said of the wellbeing manager at her centre: “…she’s absolutely fabulous. She’ll give you everything that you need. I think, having her is a major [positive] step in this whole situation, because she’s constantly sending out things, just trying to make everyone feel better. She is such a star to the business.” Multiple participants said they had good managers who routinely checked in on them, and provided support if needed. Organisations also allowed staff to adapt their working patterns and shift to accommodate their needs, such as childcare, during lockdown. This was an important organisational adaptation as childcare was cited as a major barrier to physical activity and reducing sedentary behaviour. Participant 9 (female, WHF, part time) described how this was done her:

“…they adapted my working pattern, I was on reduced hours, non-phone work for the majority of the time…I couldn’t really do phone work with the six year old around. But yeah, they’ve been really good to be honest, they’ve been really accommodating.”

During the UK lockdowns, many organisational barriers to reducing sedentary behaviour and increasing physical activity (such as the need to always be at one’s desk) remained, which, in combination with barriers at other levels of influence, made it especially difficult for participants to sit less and/or move more. Some participants also felt these barriers were shifting (ex. having more control over breaks when working at home), while others identified new barriers (ex. having back-to-back virtual meetings).

### Organisational support required

When asked what support participants might want to increase their physical activity and decreased their sedentary behaviour, participants found it difficult to pinpoint specific requests. To support increasing physical activity behaviour, a few said that stretches or lockdown friendly physical activity suggestions would be helpful. One participant said that information on physical activity targets and health requirements for their age group would be helpful to know. To support decreasing sedentary behaviour participants said having better, comfier chairs, wireless headsets, standing desks and the ability to stand in meetings generally would be helpful. Participants also requested for support relating to wellbeing and healthier lifestyles. For example, one participant said call handlers would benefit from having training on how to handle difficult calls and reduce the risk of them having a negative impact on their mental health. Another participant welcomed support on healthy eating, and how not to overeat. A few participants noted that having limited social interactions during lockdown was difficult, and any support to help with this would be beneficial. Despite these suggestions, many participants emphasised that their organisations where doing the best they could at the time (during the pandemic). For example Participant 26 (female, onsite) said, “They’ve done their best in a really difficult situation.”

## Discussion

The current study provides new insight into how changes to work related to COVID-19 influenced physical activity and sedentary behaviour in contact centres for both homeworkers and those that remained in the office. We found that contact centre staff experienced increases in sedentary behaviour and moderate decreases in physical activity levels due to a number of individual, social, environment and organisational factors. Organisational support for physical activity and the reduction of sedentary behaviour was present but varied between centres. As such, we have described possible methods for how contact centres might address the identified barriers and support needs flagged by participants (see implications for practice section below).

Our investigation adds to existing research, which indicates that contact centre staff have poorer physical and mental health outcomes due organisational and environmental barriers which existed before the pandemic [22, 23, 25]. Our findings suggest that the pandemic may have exacerbated barriers specific to physical activity and (reducing) sedentary behaviour, alongside the addition of COVID-19 specific barriers and facilitators at these ecological levels. For example, contact centres are traditionally productivity focused where staff are seated all day [14]. During the COVID-19 pandemic, some participants found that centres were busier (often due to higher call volumes) which increased the need for productivity and reduced opportunities for breaks and incidental movement. This supports existing data which cites increased call volumes across UK contact centres in the telecoms and financial services sectors [43] and across Scottish contact centres [44] during the pandemic. Breaks were also often too short. However, some homeworking participants felt they had more autonomy over their breaks and the introduction of flexible working policies in some organisations which facilitated break-taking and physical activity during the workday. New barriers also emerged, such as a lack of organisational support for homeworking setup, back-to-back meetings, lack of opportunities and reasons to break up sitting (in office and at home), closure of sport/fitness facilities, and lack of a commute (if homeworking).

Participants cited several barriers to physical activity and reducing sedentary behaviour at the individual and social levels which existed at a population level before COVID-19. Some of these barriers may have also been made worse due to the pandemic. For example, lack of perceived time and motivation as well as mental health challenges are often cited as barriers to physical activity participation and sedentary behaviour reduction regardless of the context [45, 46]. Findings revealed that the restrictions on social and physical opportunities related to physical activity and breaking up sedentary behaviour during the pandemic reduced participant’s motivation to sit less and move more, which may have been further exacerbated by reduced social opportunities (new barrier) and mental health challenges brought on by the COVID-19 situation on the whole. Other studies conducted during the pandemic also cited opportunity-related motivation as a barrier to physical activity [47, 48]. Lack of time is another commonly cited barrier to engaging in physical activity and breaking up sitting [45, 49] which participants attributed to COVID-19 related changes. For example, they felt that the busyness of work, the length of breaks, and having children at home meant that they had no time to be active during or outside work hours. Lack of time was cited as a barrier to physical activity in one study conducted during the pandemic (13% of respondents) [47], however another study found those citing lack of time decreased by 60% from pre-lockdown to during the first UK-wide lockdown [50]. New barriers included social pressure to follow the UK’s COVID-19 rules (which were strict when the data was collected), having to primarily communicate with those outside your household online and a disinterest in the limited physical activity options which were allowed at the time. Some organisations increased levels of informational support and organised virtual social activities which facilitated sedentary behaviour reduction and physical activity participation at the individual and social levels.

As mentioned, there were a mix of new and existing (and exacerbated) barriers to sitting less and moving more. Several of these (such as desk setup, length of incidental movement, becoming overly engaged in work) were linked with working from home, which was new to most participants. Although many organisations shifted to homeworking arrangements during the pandemic, many others did not, or only did so for certain staff on certain days. Furthermore, there is mixed evidence to suggest that homeworking is ‘here to stay’ as post-pandemic, organisations seem to have differing preferences [51]. As such, it is important to have an understanding of the factors related to both in-office and homeworking, and the interaction between employees working in both settings. Though COVID-19 is now endemic, many of the barriers to physical activity and (reducing) sedentary behaviour may still be relevant, in particular those which were pre-existing or relate to working from home. These include 1) individual barriers (such as being overly engaged in work, the perceived need to always be contactable and lack of motivation), 2) social barriers (such as online communication for work), 3) environmental barriers (such as lack of incidental movement if working from home, poor homeworking setup, blending of the home and work environment, and not having a commute) and 4) organisational factors (such as workload, back-to-back virtual meetings, and length of breaks). Furthermore, many health and safety restrictions (such as handwashing) have not gone away and there is always the possibility of a future pandemic, in which case all the identified barriers will become very relevant again. New innovative strategies are needed to adapt to the combination of novel and well-known barriers which have occurred in this new (hybrid) working context and to be prepared should another pandemic arise. We have indicated some suggestions below which we believe will help contribute to this goal.

### Implications for practice

Using the barriers, facilitators and suggested organisational support identified in this paper as a guide (see Table 3), contact centres would benefit from speaking to staff on a routine basis to understand the most influential barriers and facilitators to physical activity and (reducing) sedentary behaviour which are specific to their organisational context. For example, participants felt that informational support (such as newsletters) was helpful, however more tangible support, such as wellbeing training or funds to purchase home working equipment is needed. They also indicated that back-to-back virtual meetings impeded their ability to take breaks, breaks were sometimes too short, and having more autonomy over their breaks and their work schedule (flexible working) was helpful. Looking to see if these barriers, facilitators and support needs are also relevant to your organisation could be a place to start.

**Table 3:** Summary of barriers and facilitators to physical activity and (reducing) sedentary behaviour in contact centres.

Ecological level	Reducing Sedentary behaviour		Physical activity	
	Barrier	Facilitator	Barrier	Facilitator
**Individual**	Sitting more due to anxiety Perceived need to always be at desk and contactable Overly engaged in work	Informational support	Lack of motivation, self-discipline, desire Going out less due to anxiety Lack of routine Lack of preferable options	Informational support
**Social**	Reduced social interaction with colleagues Social pressure to follow rules Online communication for work (even if in-office)	Online communication for work (even if in-office)	Inability to meet with those outside household Fear of crowded spaces Children at home/spending time with family	Children at home/spending time with family
**Environmental**	Reduction in instances/length of incidental movement (onsite/WFH) Lack of opportunities to leave desk Poor office setup (WFH) Blending of home/work environment (WFH)	Ability to break up sitting by completing housework	Reduction in instances/length of incidental movement (onsite/WFH) Closure of sport/fitness facilities No commute Built environment at home or near office	Built environment at home or near office
**Organisational**	Busier at work, tied to phone Back-to-back virtual meetings Lack of support for homeworking setup (WFH)	Support for homeworking setup (WFH) More autonomy over breaks (WFH) Wellbeing manager Flexible working to accommodate individual needs	Busier at work, tied to phone Breaks too short Back-to-back virtual meetings	More autonomy over breaks (WFH) Wellbeing manager Flexible working to accommodate individual needs

### Implications for research

Future research should examine the long-term effects of hybrid and remote working on physical activity and sedentary behaviour in contact centres and other desk-based working environments, incorporating economic factors. For example, studies could investigate how economic pressures, such as increased productivity demands and cost-cutting measures, influence organisational support for physical activity and reducing sedentary time. The cost-effectiveness of interventions like flexible working policies, wellness programmes, and ergonomic home office setups should also be explored. Additionally, future research should identify interventions suited to both home and office environments and explore the role of technology in promoting movement. Understanding how individual factors like mental health and motivation interact with organisational, environmental, and economic conditions will be key to developing effective interventions.

### Strengths and limitations

This study contributes novel insight into changes to physical activity and sedentary behaviours for contact centre workers during the UK COVID-19 lockdowns from a rigorous qualitative analysis. Data was collected from four contact centres of varying size and sector, including staff who worked from the office and from home. The insights gained through the study link pre-existing barriers to new ones that have emerged since the onset of the COVID-19 pandemic through the lens of the ecological model, which allowed for in depth understanding of barriers at all levels of influence.

As participants were partially recruited via convenience sampling, it is possible that they may have already been interested in speaking about physical activity and sedentary behaviour and therefore have been experiencing less or different barriers and facilitators to participating in these behaviours.

## Conclusions

The findings from this study highlight a number of barriers to physical activity and (reducing) sedentary behaviour for contact centre staff, alongside facilitators and suggestions for how to address them in the workplace (wherever staff are working from). Although it was a UK based study, many of the barriers would be applicable to contact centres worldwide. It is necessary for contact centre organisations to consider these insights and their relevance to their specific organisation in order to support and/or improve physical activity and the reduction of sedentary behaviour levels. The findings from this study might benefit other types of organisations that employ desk-based workers as the identified barriers, facilitators and support needs may also be relevant. Future studies or policies and practices should focus on developing effective and sustainable solutions through addressing the identified barriers which are relevant to an organisation’s culture and context.

## Supporting information

S1 File COREQ checklist. (DOCX)

S2 File Participant characteristics. (DOCX)

S3 File Interview topic guide. (DOCX)
