# Supplementary material for: Factors influencing physical activity and sedentary behaviour in contact centres during the COVID-19 pandemic and their relevance for the future of hybrid working
Source: PLoS One. 2024 Oct 23;19(10):e0312473. doi: 10.1371/journal.pone.0312473 (PMC11498657; doi:10.1371/journal.pone.0312473)
Supplement: S2 File — (DOCX) [file pone.0312473.s002.docx]

| **Participant id** | **Gender** | **WFH/Onsite** | **Part time/Full time** |
| --- | --- | --- | --- |
| **Centre A (private)** | | | |
| P1 | Female | Onsite | Part time |
| P2 | Male | Onsite | Full time |
| P3 | Male | Onsite | Full time |
| P4 | Male | Onsite | Part time |
| P5 | Male | Onsite | Undisclosed |
| P6 | Male | Undisclosed | Undisclosed |
| P7 | Male | WFH | Full time |
| P8 | Male | WFH | Full time |
| **Centre B (public)** | | | |
| P9 | Female | WFH | Part time |
| P10 | Female | WFH | Full time |
| P11 | Female | WFH | Full time |
| P12 | Male | WFH | Full time |
| P13 | Male | WFH | Full time |
| P14 | Male | WFH | Full time |
| P15 | Male | Undisclosed | Undisclosed |
| P16 | Female | WFH | Full time |
| P17 | Male | WFH | Full time |
| P18 | Female | WFH | Full time |
| P19 | Female | WFH | Part time |
| **Centre C (public)** | | | |
| P20 | Female | WFH | Full time |
| P21 | Female | Onsite | Undisclosed |
| P22 | Male | WFH | Full time |
| P23 | Female | WFH | Undisclosed |
| P24 | Female | Onsite | Full time |
| P25 | Male | Onsite | Undisclosed |
| P26 | Female | Onsite | Undisclosed |
| P27 | Female | Onsite | Part time |
| **Centre D (private)** | | | |
| P28 | Male | WFH | Undisclosed |
| P29 | Female | WFH | Full time |
| P30 | Female | Undisclosed | Undisclosed |
| P31 | Female | WFH | Full time |
| P32 | Female | Onsite | Undisclosed |
| P33 | Male | Undisclosed | Undisclosed |

*WFH = participant was working from home
