# Supplementary material for: Factors influencing physical activity and sedentary behaviour in contact centres during the COVID-19 pandemic and their relevance for the future of hybrid working
Source: PLoS One. 2024 Oct 23;19(10):e0312473. doi: 10.1371/journal.pone.0312473 (PMC11498657; doi:10.1371/journal.pone.0312473)
Supplement: S3 File — (DOCX) [file pone.0312473.s003.docx]

**Current work conditions**

1. Where are you currently working from?

- From home- full time
- From home- part time
- From the office- full time
- From the office- part time

1. Tell us more about your work environment:

[If working from home]

What issues are you facing with home working? Please mention any personal, job-related, environmental, or social issues you may be facing while working from home.

[If going to work]

How have things changed at work during the COVID pandemic? Please mention any personal, job-related, environmental, or social issues you may be facing currently at work.

**Sedentary behaviour: This relates to sitting behaviour**

1. Are your levels of sedentary behaviour more, less or the same as before the pandemic?
2. What are some of the current barriers you face relating to sitting behaviour? Please mention any personal, job-related, social or environmental barriers.

**Physical activity: this relates to any light, moderate or vigorous activity you may be doing. Example: walking, exercise class, swimming**

1. Are your levels of physical activity more, less or the same as before the pandemic?
2. What are some of the current barriers you face relating to being more active? Please mention any personal, job-related, social or environmental barriers.
3. Has the way you travel to work and other places been affected by the pandemic? Please elaborate.
4. Has the current work situation helped in any way to sit less and move more? Why?

**Other factors**

1. Do you smoke? -Yes, No
2. If Yes --> Has the pandemic altered your smoking patterns? Please elaborate.
3. Has the pandemic had any effect on your eating behaviour? Please elaborate.
4. Are you currently isolating?
5. Do you have caring responsibilities?

**Support**

1. Are you keen to make changes to your PA and/or SB levels?
2. What support, resources or tools do you think might help you sit less and move more?
